# Analysis of bacterial diversity and functional differences of Jiang-flavored Daqu produced in different seasons

**DOI:** 10.3389/fnut.2022.1078132

**Published:** 2023-01-04

**Authors:** Lamei Wang, Yuxin Cheng, Xiaoxia Hu, Yongguang Huang

**Affiliations:** ^1^Key Laboratory of Fermentation Engineering and Biological Pharmacy of Guizhou Province, College of Liquor and Food Engineering, Guizhou University, Guiyang, Guizhou, China; ^2^Guizhou Moutai Brewery (Group) Xijiu Co., Ltd., Xishui, Guizhou, China

**Keywords:** Jiang-flavored Daqu, different seasons, bacterial community structure, functional bacterial genera, metabolic pathway

## Abstract

High-temperature Daqu is an important saccharifying fermenting starter for brewing Jiang-flavored Baijiu. This paper analyzed the diversity characteristics of bacterial communities of Jiang-flavored Daqu (JFDQ) with seasonal changes through Illumina HiSeq sequencing and multivariate statistical methods. Results showed that 21 phyla, 529 genera, and 47 core bacterial genera were identified from the 48 composite samples. Among them, eight functional genera were only found in the summer-produced Daqu (*Propionigenium*, etc.). *Pantoea*, *Bacillus*, *Lentibacillus*, and *Oceanobacillus*, respectively, served as the representative functional bacterial genera of the four seasons. Functional prediction analysis showed that Amino acid metabolism Carbohydrate metabolism, Lipid metabolism, Metabolism of cofactors and vitamins, and Nucleotide metabolism (relative abundance > 1%) were the most critical microbial functions in JFDQ, and these key enzymes involved in acetoin biosynthesis, and acetyl-CoA biosynthesis were more abundant in the summer than in the winter. The functional microorganisms community in this paper would provide valuable suggestions about the seasonal production of JFDQ, guiding the Baijiu brewing processes.

## 1 Introduction

**FIGURE 1:**
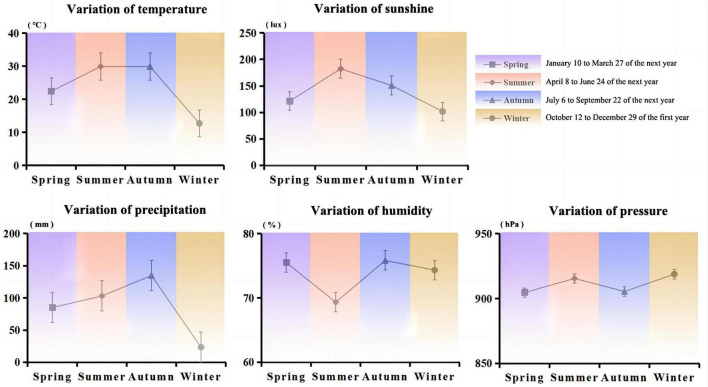
Variations in the average values of five climatic factors (temperature, sunshine, precipitation, humidity, and pressure) for different seasons in Moutai Town, Guizhou Province.

Jiang-flavored Baijiu, especially Moutai, is produced at Moutai town (27°81′ N; 106°41′ E) on the banks of the Chishui River in Guizhou Province, with an average annual temperature of 10–30°C, the sunshine of 100–185 lux, the humidity of 60–90%, precipitation of 20–135 mm, and pressure of 900–920 hPa. Moutai town is typical for microbial growth in the brewing production process under the unique geographical environment and climate conditions (Figure 1) (1). High-temperature Daqu is a saccharifying fermenting starter for brewing Jiang-flavored Baijiu, and the entire production, as well as storage processes, are completely exposed to ambient air. During Daqu fermentation, many microorganisms are enriched from the natural environment, forming complex microbial resources as well as producing abundant enzyme and flavor compounds, especially the functional microbiota (Relative abundance > 1%, and they metabolize flavor compounds and hydrolases in Daqu) in Daqu. For example, the Daqu is rich in bacteria, mainly including *Bacillus*, *Clostridium, Lactobacillus*, etc., which are key contributors to Baijiu brewing (2, 3). Moreover, the functional microbial communities play a major role which can metabolize a variety of enzymes to produce rich Baijiu aroma substances along with their precursors and improve the flavor structure of the Baijiu (4–6).

At present, based on the important functional role, many studies have focused on the microbial community structure of Jiang-flavored Daqu by culture-dependent and independent methods (7–9), but there are some gaps in the research on the differences of microbial community composition with time succession. The fermentation process and quality of Daqu are influenced by complex factors during fermentation, and the microbial communities inside Daqu are affected by biotic and abiotic factors in their habitats (10). For example, a large number of microorganisms from the environment are screened and enriched into Daqu during production. Moreover, there are obvious differences in microbial flora in different seasonal environments. Research showed that the microbial diversity of the Chishui River was richer in summer than that in the other three seasons (11), indicating that the environmental climate conditions in different seasons have a great impact on microbial ecology. Additionally, there were differences in the saccharification, fermentation, and aromatization abilities of Daqu produced in different seasons (12–16). While the specific mechanisms and causes of the differences in the quality of Daqu production with different seasons have not been reported in detail.

The changes in environmental conditions in different seasons are highly responsible for the changes in the microbial structure of Daqu during the production process. For example, there were differences in the microbial communities of Tao-Hua qu in spring and summer, with six to eight folds more prokaryotic microorganisms than eukaryotic microorganisms (17). The number of microbiota in spring and summer Bran Cuqu were higher than those in autumn and winter Bran Cuqu (18). Accordingly, the microbial community structure of Daqu is closely related to the brewing microecological environment, and seasonal changes are one of the main factors affecting the microecology of the brewing environment. At present, there is insufficient research on Daqu with different seasons. Therefore, it’s necessary to study the effects of seasonal changes on the microbial community structure of Jiang-flavored Daqu, especially functional microbes. For example, bacteria not only provide a rich enzyme system during the fermentation process but also produce a rich body of flavor compounds and their precursors (2, 3). Understanding the microbial succession with different seasons and their functional bacteria may contribute to Daqu production under controlled conditions.

In this study, the succession and characteristics of bacterial structures in the production of Daqu in different seasons were investigated. The microecological differences, the structure of the functional genus, and their brewing function were further analyzed to reveal the mechanisms of JFDQ produced in different seasons. Then, the functional profiles and key enzymes of JFDQ bacteria between different seasons were demonstrated. This study will provide scientific theory and data support for the further development of traditional and modern Daqu production throughout the year.

## 2 Materials and methods

### 2.1 Sample collection

Samples were collected from 17 representative brewing enterprises in the Chishui River basin of Guizhou Province, including Guanyinsi district, Shangping village, Yantan village, etc. in Moutai town. Samples were designated as: winter (period of production from October 12 to December 29 of the first year); spring (period of production from January 10 to March 27 of the next year); summer (period of production from April 8 to June 24 of the next year); and autumn (period of production from July 6 to September 22 of the next year). All samples were produced by the same Jiang-flavored Daqu production process: (i) shaping, (ii) fermentation (for about 40 days), and (iii) storage (for about 6 months). Additionally, a block of Daqu was selected from the constant upper, middle, and lower locations in the representative brewing enterprise’s storage room. After crushing and mixing, take 250 g of each block of Daqu, and then take 100 g of each of the three samples for uniform mixing. The 17 representative enterprises are divided into 7 areas, and equal amounts of samples from the same area were mixed as one experimental sample. Twelve samples were collected in each quarter according to the period of production, and a total of 48 composite samples were collected in four seasons (118 Daqu samples before mixing well). The samples were stored at -80°C for 24 h before DNA extraction.

### 2.2 DNA extraction and PCR amplification

To obtain the genomic DNA of the microbial community, the 48 Daqu samples were extracted using an E.Z.N.A. Soil DNA Kit (Omega Bio-Tek, USA) according to the manufacturer’s instructions. The 338F (5′-ACTCCTACGGGAGGCAGCAG-3′) and 806R (5′-GGACTACHVGGGTWTCTAAT-3′) were applied to amplify the 16S rRNA gene of the bacterial V3-V4 variable region. The amplification procedure was as follows: 95°C predenaturation for 3 min, 27 cycles of 95°C denaturation for 30 s, 55°C annealing for 30 s, and 72°C extensions for 30 s, and 72°C extensions for 10 min. The amplification system was as follows: 4 μl 5 × FastPfu buffer, 2 μl 2.5 mmol/L dNTPs, 0.8 μl primers (5 μmol/L), 0.4 μl FastPfu polymerase, and 10 ng DNA template. The PCR products were detected on 2% (w/v) agarose gels, and the size of the paired-end sequence was no less than 550 bp. Subsequently, the PCR products were analyzed via the Illumina MiSeq 300 platform (San Diego, CA, USA).

### 2.3 Climatic factors

The collection and measurement of climatic factor values were carried out by our lab and the Guizhou Meteorological Bureau, including temperature, sunshine, precipitation, humidity, and pressure.

### 2.4 Sequence and statistical analysis

The raw data were filtered to remove joints and low-quality sequences through Quantitative Insights into Microbial Ecology (QIIME v1.8.0). Then operational taxonomic units (OTUs) were clustered with a threshold of 97% sequence similarity by UPARSE software (version 7.1), and Chao 1 and Shannon indices were calculated using Mothur.^1^ Bacterial community composition mapping was performed using Majorbio,^2^ Principal component analysis (PCA) and linear discriminant analysis effect size (LEfSe) analysis were carried out to evaluate significant differences (LDA > 3, *P* < 0.05) in the structural composition of bacteria. The abundance bubble map and redundancy analysis (RDA) were plotted by the “ggplot 2” and “vegan” packages of R software (v 4.1.1), separately. While principal coordinates analysis (PCoA, K ≥ 2) of the microbiota typing prediction was plotted by the “cluster” package. The potential function of the bacterial community was predicted using the PICRUSt2 software, and enzymes encoded by predicted functional genes were explored based on the KEGG database information.

## 3 Results and discussion

### 3.1 The composition of the bacterial community

**FIGURE 2:**
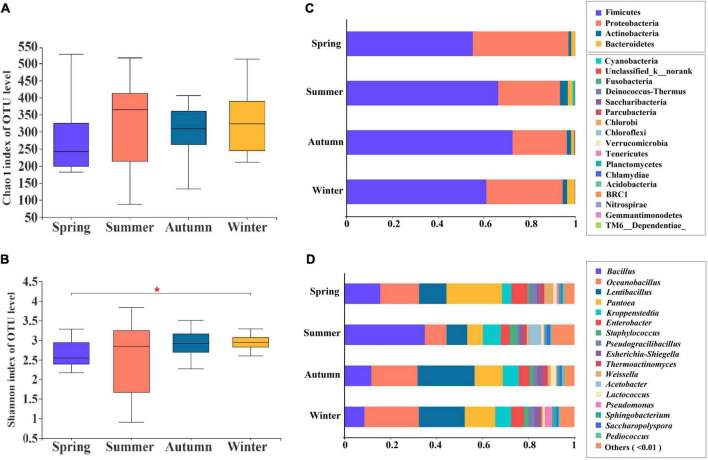
Chao 1 **(A)** and Shannon **(B)** indices of bacterial α-diversity, and the bacterial community structure at the level of phylum **(C)** and genus **(D)** in different seasons. *P < 0.01.

Significance of changes in α-diversity, as measured by the Chao 1 (Figure 2A) and Shannon indices (Figure 2B), were analyzed to identify the bacterial community richness and diversity indices (Supplementary Table 1) in JFDQ produced in four seasons on the OTU level. The Chao 1 index of summer-produced Daqu was higher in comparison with the other seasons (*P* < 0.05), indicating that the bacterial species in summer-produced Daqu were the most abundant and that the diversity feature was obvious in four seasons. In summer-produced Daqu, the Shannon index (Figure 2B) was larger, and its dispersion was also greater than that in winter-produced Daqu. Meanwhile, the bacterial abundance in summer-produced Daqu was higher and more uniformly distributed than that in autumn-produced Daqu (12, 19). Additionally, significant differences (*P* < 0.05) were observed in microbial diversity among the four seasons, with the highest microbial diversity in summer and the lowest diversity in winter. The results indicated that the species distribution was more uniform and the microecosystem was more stable in the summer-produced Daqu in comparison with the remaining three seasons, especially winter, which illustrated the reason for the generally high quality of the summer-produced Daqu.

A total of 1,382 OTUs were obtained from 48 composite samples, and 21 phyla and 529 genera were identified, excluding sequences that could not be effectively compared. Rarefaction curves and Shannon curves based on the OTUs of 97% similarity tended to reach saturation (Supplementary Figure 1), indicating that our sequencing depth met the requirements for sequencing and analysis. The bacterial community structural composition of JFDQ produced in four seasons was shown in Figure 2C. Four major dominant phyla had an average relative abundance greater than 1% in four seasons, respectively, which were Firmicutes (57.04, 67.48, 74.99, and 66.47%), Proteobacteria (40.30, 25.55, 21.73, and 28.59%), Actinobacteria (1.18–3.30%), and Bacteroidetes (1.22–2.91%). Moreover, seventeen phyla with an average relative abundance of less than 1% were identified (Cyanobacteria, Fusobacteria, Saccharibacteria, Pacuribacteria, Chloroflexi, etc.). In the previous studies of our lab, Firmicutes, Proteobacteria, and Actinobacteria served as the main dominant phyla from mechanically pressed Jiang-flavored Daqu (16) and mechanical brewing stacked fermentation spirits (20). In addition, Bacteroidetes was detected as one of the main dominant phyla from brewing environments in Moutai town (21). Moreover, the low abundance of Bacteroidetes in Daqu was significantly enriched in the environment, which tentatively indicates that diffusion and migration of this phylum may occur between the brewing environment, Daqu, and stacked fermentation spirits. Thus, a comparative analysis of the abovementioned studies and the results of this study revealed that the production of Daqu in different seasons was significantly correlated with the brewing environment and stacked fermentation spirits in Moutai town.

Furthermore, a total of 529 genera were detected in the JFDQ samples. As shown in Figure 2D, the 17 dominant bacterial genera with an average relative abundance greater than 1% were identified, and the genus structure indicated that there were significant differences in the distribution of bacterial genera among the four seasons. For example, the average relative abundance of *Bacillus* in summer-produced Daqu was 34.91 ± 0.20%, significantly higher than that in spring (15.50 ± 0.07%), autumn (11.65 ± 0.03%), and winter (8.64 ± 0.04%), suggesting high-temperature is the main reason for the growth of *Bacillus*, which is consistent with Feng, Lu (2). The results showed that *Bacillus* played an important role in the formation of sauce flavor in the production of sauce flavor Baijiu (22, 23). The average relative abundance of *Oceanobacillus* in summer-produced Daqu was 9.45 ± 0.04%, which was significantly lower than that in spring (16.93 ± 0.09%), autumn (20.08 ± 0.09%), and winter (23.65 ± 0.13%). In addition, *Oceanobacillus* was also detected in the brewing environment (21) and stacked spirits (20) of Moutai town. The high-temperature environment was unfavorable for the growth of *Oceanobacillus* (12). The average relative abundance of *Lentibacillus* and *Pantoea* contained in summer-produced Daqu were 8.99 ± 0.04 and 6.81 ± 0.03%, respectively, which were significantly lower than that in spring (11.89 ± 0.04, 24.14 ± 0.13%), autumn (24.81 ± 0.06, 12.29 ± 0.09%) and winter (19.98 ± 0.06, 13.29 ± 0.10%). Consistent with the study of Zuo, Huang (16), the average relative abundance of *Lentibacillus* and *Pantoea* decreased to less than 1% at the first turning of the brick, suggesting that the temperature of Daqu is the main factor affecting the production of *Lentibacillus* and *Pantoea* in different seasons. The data showed that *Kroppenstedtia* had a high average relative abundance (7.81 ± 0.01%) in summer-produced JFDQ, indicating that *Kroppenstedtia* had high-temperature tolerance properties. *Kroppenstedtia* can produce protease with well-developed mycelium, which can enhance the protease activity of daguerreotypes and promote the internal structural loosening of Daqu (24). The average relative abundance of *Enterobacter* in JFDQ of four seasons did not change much (3.81–6.98%). *Enterobacter* was also detected in the air microorganisms (25), Jiang-flavored Daqu, and fermentation spirits (26) in Moutai town. Furthermore, *Enterobacter* was more abundant in the brewing environment than the Daqu in comparison with the study of Ren, Huang (27), suggesting that *Enterobacter* is stably present in the brewing process of Baijiu, and may originate from the brewing environment. The above results showed that the main dominant bacterial genera were significantly enriched in summer-produced JFDQ, which fully illustrated the advantage of producing JFDQ in the high-temperature season and the selective contribution mechanism of high temperature. In particular, the dominant bacteria play a major contributing role in the brewing process of Baijiu.

### 3.2 The difference in the bacterial community

**FIGURE 3:**
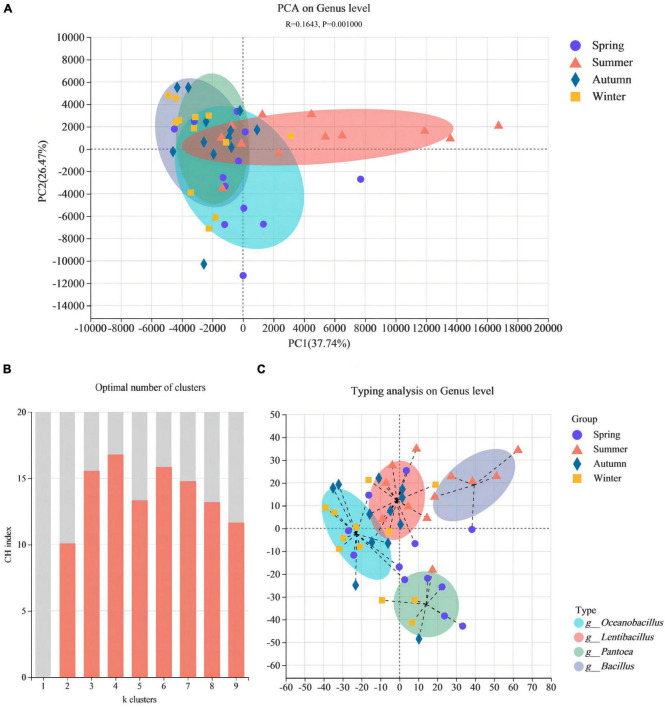
PCA shows the bacterial β-diversity in different seasons **(A)**. Maximum CH index **(B)** to calculate optimal clustering *K* values (*K* ≥ 2) for microbiota typing prediction in different seasons, determined via PCoA **(C)**.

Based on the genus level, PCA (ANOSSIM, *R* = 0.1643, *P* = 0.001) was performed to further reveal the β-diversity in bacterial community structure between different JFDQ production seasons, the results were shown in Figure 3A. The bacterial communities of JFDQ produced in spring, autumn, and winter were randomly distributed in the second and third quadrants. Although a certain distance was observed between the three, they were farther from the summer-produced JFDQ. Accordingly, the bacterial community structure of JFDQ produced in the four seasons was similar, but the bacterial community structure of summer-produced JFDQ was different from the other three seasons. It was inferred from this data that the main reason for this difference in microbial structure was the effect of seasonal temperature. Meanwhile, previous studies showed that differences in the variety and quantity of microorganisms in the raw materials, equipment, site, and air in the production process were caused by high temperature and humidity in summer, which is conducive to the enrichment of beneficial microorganisms in the Daqu bricks (9, 28). In addition, the microbiota typing (Figures 3B, C) was predicted based on the relative abundance of the microbiota at the genus level by the PCoA (Jensen-Shannon Distance, K ≥ 2). The result of Figure 3C showed that four dominant genera with an average abundance greater than 0.1% were derived based on 48 composite samples, namely *Pantoea*, *Oceanobacillus*, *Lentibacillus*, and *Bacillus*.

### 3.3 The characteristics of the dominant and functional bacterial community

**FIGURE 4:**
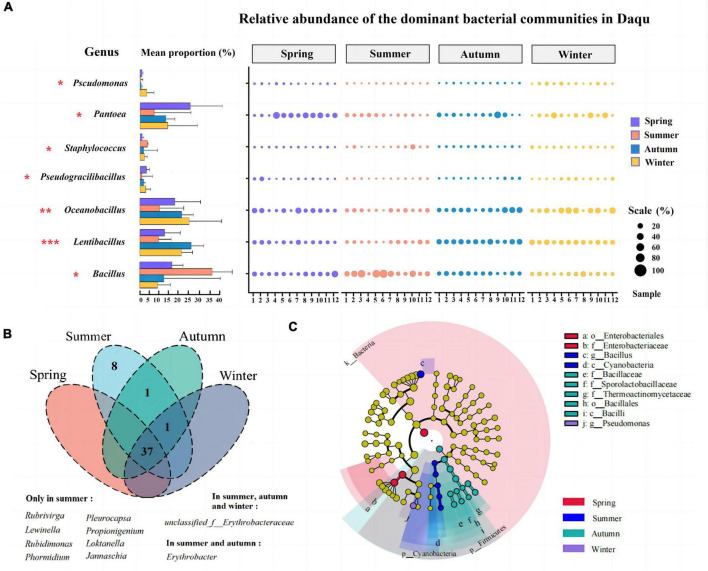
Changes and significant differences of the dominant bacterial genera (>0.1%) and the core bacterial genera (>1%) in the 48 Daqu samples produced in four seasons, as determined via the Kruskal-Wallis H test bar plot and abundance bubble map **(A)**, venn **(B)**, LEfSe **(C)**. *P < 0.05, **P < 0.01, ***P < 0.001.

Furtherly, differences in the abundance of bacterial genera in the 48 composite samples were performed by the Kruskal-Wallis H test (One-way ANOVA, *P* < 0.05). The left results of Figure 4A indicated that there were seven bacterial genera with an average abundance greater than 0.1% significant differences in the community structure of JFDQ (*Pseudomonas*, *Pantoea*, *Pseudogracilibacillus*, *Staphylococcus*, *Oceanobacillus*, *Lentibacillus*, and *Bacillus*), which revealed the similarity and differences in the dominant bacterial composition of JFDQ produced in different seasons. As shown on the left of Figure 4A, *Pseudomonas*, *Staphylococcus*, and *Pseudogracilibacillus* showed significant variation (*P* = 0.034, *P* = 0.027, and *P* = 0.045) with the season. *Pseudomonas* can synthesize phenazine during Daqu fermentation (29). *Staphylococcus* has been detected in soybean, strong-flavored Daqu, and various traditional fermented foods (27, 30), but its role has yet to be explored in-depth (31, 32).

In addition, the results of Figure 4A showed that *Pantoea* (*P* = 0.018), *Bacillus* (*P* = 0.019), *Lentibacillus* (*P* < 0.001), and *Oceanobacillus* (*P* = 0.009) were extremely significantly enriched in spring, summer, autumn, and winter-produced JFDQ, respectively. *Pantoea* was an important chemo energetic heterotrophic bacterium with metabolic and fermentation types, which has been reported several times in the brewing industry (7). *Bacillus* has been extensively studied in various types of Baijiu, and *Bacillus* is the main functional bacterium involved in the fermentation of Baijiu (22, 33, 34). *Bacillus* can secrete hydrolytic enzymes, such as amylase and protease, which can convert macromolecules (including starch and protein) into glucose and amino acids, which in turn produce a variety of flavor precursors and substances. At the same time, the free amino acids produced by the metabolism of *Bacillus* through the Melad reaction add a unique sauce flavor that contributes to the flavor formation of Baijiu. *Oceanobacillus* plays an important role in the production of enzymes such as protease, amylase, cellulase, and esterase during the fermentation of Chinese Baijiu (33). *Oceanobacillus* in the autumn-produced Daqu was richer than that in the summer-produced Daqu. Moreover, *Oceanobacillus* contributed to the enhancement of the esterification and saccharification power of Daqu (12). Therefore, the results of the Kruskal-Wallis H test suggested that these four bacterial genera could be considered as the representative functional bacterial genera for the four seasons, respectively. The above analysis furtherly explained the reasonable mix of Daqu produced in different seasons through making full use of the functions of the dominant bacteria can ensure consistent quality while also enhancing the mellowness and aging flavor of the base wine.

Bacterial genera with an average relative abundance greater than 1% in the samples and present in at least one sample were defined as core microorganisms (35, 36). Forty-seven core bacterial genera were statistically analyzed in the 48 composite samples (Figure 4B), and thirty-seven core bacterial genera were present in four seasons of JFDQ. Among them, eight genera were only found in the summer-produced Daqu, namely *Rubrivirga*, *Lewinella*, *Phormidium*, *Pleurocapsa*, *Propionigenium*, *Jannaschia*, *Rubidimonas*, and *Loktanella*. Among them, *Propionigenium* can be fermented and metabolized to produce propionic acid (37), which increases the types of acids in Baijiu. Propionic acid is tart and sweet, and a small amount can make the wine softer. *Loktanella* can produce natural red pigment (38), and it is speculated that this genus has a great relationship with the production of Hongxing Daqu. *Erythrobacter*, a widely studied marine bacterium that can degrade substances and produce various carotenoids (39), was only detected in summer and autumn-produced Daqu. Furthermore, LEfSe (LDA > 3, *P* < 0.05) was performed to evaluate significant differences in the core bacteria of JFDQ produced in four seasons. Thirteen differential taxa were significantly enriched among the 47 core bacteria (Figure 4C). At the family level, Enterobacteriaceae enriched in spring-produced JFDQ, while Thermoactinomycetaceae and Sporolactobacillaceae enriched in autumn-produced JFDQ. Enterobacteriaceae are involved in the formation of esters in Baijiu (31). At the genus level, *Bacillus* and *Pseudomonas* enriched in summer and winter-produced JFDQ, respectively. *Bacillus* has a strong tolerance to temperature and can secrete hydrolytic enzymes and produce pyrazine substances through metabolism, thus improving the flavor of Baijiu, which is the reason for the better quality of high-temperature Daqu (23, 40). *Pseudomonas* is a new strain with biological control function, which can secrete secondary metabolites phenazines with broad-spectrum antibacterial activity (41). Accordingly, we conclude that these taxa are considered to be the representative functional bacterial genera for each season.

### 3.4 Prediction of functional composition of bacterial community

**FIGURE 5:**
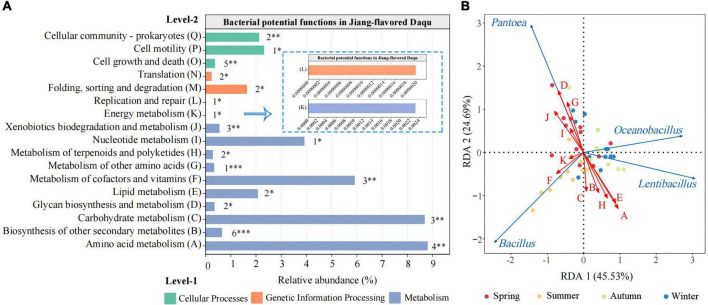
An overview of the bacterial functions in Daqu predicted by PICRUSt2 based on 16S rRNA sequencing data **(A)**. RDA biplot displaying the relationship between the differential metabolic pathways and dominant bacterial genera **(B)**. Red letters represent differential metabolic pathways, and the blue arrows represent dominant bacterial genera. Significant difference is represented by *(*P* < 0.05), **(0.001 ≤ *P* < 0.01), ***(*P* < 0.001).

To further understand the effects of the bacteria in Daqu, this study used PICRUSt2 to predict their potential functions based on 16S rRNA sequencing data. As shown in Figure 5A, a total of 41 different level 3 KEGG metabolic pathways (Supplementary Table 2) were predicted and screened (*P* < 0.05) in Daqu samples from four different seasons. They were divided into three categories (level-1), namely cellular processes, genetic information processing, and metabolism. The level-1 main functional group was metabolism which exhibited the highest relative abundance, signifying the presence of vigorous microbial metabolism in JFDQ samples. Meanwhile, the following five level-2 functions were dominant (relative abundance > 1%), including Amino acid metabolism (A), Carbohydrate metabolism (C), Lipid metabolism (E), Metabolism of cofactors and vitamins (F), and Nucleotide metabolism (I), suggesting that they were the most critical microbial functions in JFDQ.

Based on the RDA, we found significant associations between different metabolic pathways and dominant bacterial genera (>0.1%) (Figure 5B). Results showed that *Bacillus* and *Lentibacillus* were consistent with Amino acid metabolism (A), Biosynthesis of other secondary metabolites (B), Carbohydrate metabolism (C), Lipid metabolism (E), and Metabolism of terpenoids and polyketides (H), while *Pantoea* significantly related to Glycan biosynthesis and metabolism (D), Metabolism of other amino acids (G), Nucleotide metabolism (I), and Xenobiotics biodegradation and metabolism (J). In addition, microbial function Amino acid metabolism (A) and Carbohydrate metabolism (C) showed the highest relative abundance (>8%) in the metabolic functions, mainly because thermophilic *Bacillus* and *Lentibacillus* enriched in summer and autumn during the Daqu-making process, metabolizing proteins to produce a variety of free amino acids (4, 6). At the same time, the enriched amino acids in the fermentation process provide nitrogen sources for microbial metabolism, promote the growth of microorganisms and the production of metabolites (42), and maintain the cycle of microorganisms and fermentation. This furtherly explained that summer and autumn are excellent for the screening and enrichment of Baijiu brewing functional microorganisms.

### 3.5 Profiles of key enzymes involved in flavor formation

**FIGURE 6:**
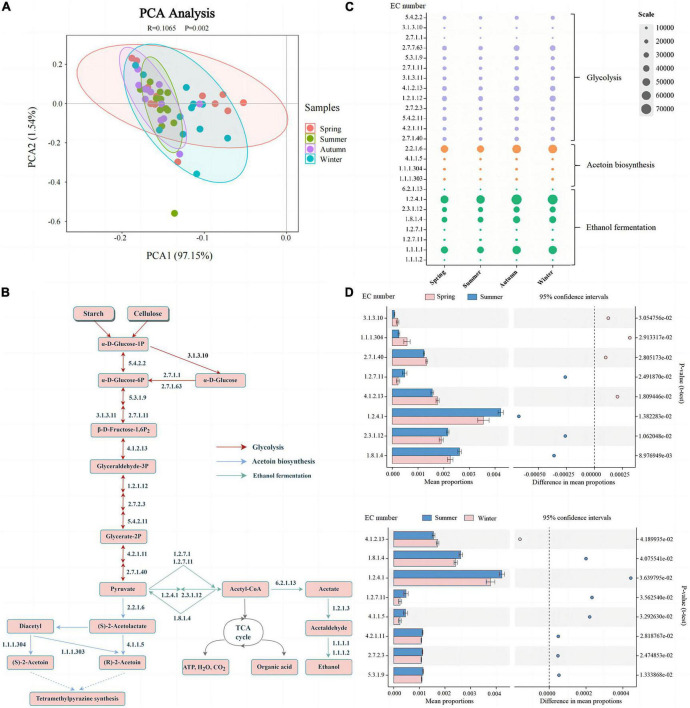
Differences in predicted enzymes of bacterial community found in Daqu samples from different seasons. PCA with the β-diversity of all the predicted enzymes **(A)**. Profile of main enzymes related to glycolysis, acetoin biosynthesis, and ethanol fermentation (acetyl-CoA biosynthesis) **(B)**. Relative abundance distribution of enzymes **(C)** shown in subplot **(B)**, and significant changes between spring and summer and summer and winter **(D)**.

The microorganisms in Daqu secrete various hydrolases related to hydrolyzing the carbon source and nitrogen source of the fermentation substrate, which promote the production of ethanol and flavor compounds (5). Therefore, we focused on the distribution of enzymes involved in glycolysis, acetoin biosynthesis, and ethanol fermentation of Daqu produced in different seasons (Supplementary Table 3). According to the result of PCA (ANOSSIM, *R* = 0.1065, *P* = 0.002), clustering showed that the functional enzymes secreted by the main microorganisms in summer significantly differed from spring and winter (Figure 6A). This furtherly suggested that the functions of microbes in summer-produced Daqu are similar to those in autumn, but different from those in spring and winter.

As shown in Figure 6B, there were 25 key enzymes related to glycolysis, acetoin biosynthesis, and ethanol fermentation pathways in the metabolic pathway. Meanwhile, the results in Figures 6C, D showed that there were significant differences in the enzymes related to glycolysis (EC 3.1.3.10, EC 5.3.1.9, EC 4.1.2.13, EC 2.7.2.3, EC 4.2.1.11, EC 2.7.1.40) between the summer-produced Daqu and those in spring and winter. Through the secretion and enrichment of these enzymes, the fermentation process of fermented grains is promoted, and starch and cellulose are hydrolyzed by α-amylase, glucoamylase, cellulase, and β-glucosidase to produce glucose. Glucose is converted into glucose-6p by hexokinase (EC 2.7.1.1), which is the first key enzyme of glycolysis. Moreover, Glucose-6-phosphate isomerase (EC 5.3.1.9) and pyruvate kinase (EC 2.7.1.40) also play a major role in the irreversible reaction of hexose to pyruvate. We also found that compared with summer-produced Daqu, the key enzymes related to glycolysis (EC 3.1.3.10, EC 4.1.2.13, EC 2.7.1.40) were significantly enriched in spring-produced Daqu, which was dominated by *Pantoea*.

During fermentation in pits, bacteria and fungi secrete alcohol dehydrogenase (EC 1.1.1.1) under anaerobic conditions, and pyruvate produces ethanol under the action of alcohol dehydrogenase (5, 6). Notably, the abundance of alcohol dehydrogenase (EC 1.1.1.1, 30.55–32.04%) was high in all four seasons, indicating that alcohol metabolism was dominant and its relative abundance was very large. In addition, pyruvate and acetyl-CoA are central compounds in major macromolecular metabolism, as well as in the anabolism of fatty acids and amino acids (43). Pyruvate is converted into acetyl-CoA under aerobic conditions, which is completely oxidized to CO_2_ and H_2_O through the TCA cycle, and various organic acids are generated simultaneously. Figures 6C, D showed that the key enzymes (EC 1.2.7.11, EC 1.2.4.1, EC 2.3.1.12, EC 1.8.1.4) involved in acetyl-CoA were significantly more enriched in summer than in spring and winter. Acetoin is an important precursor for the synthesis of tetramethylpyrazine, and the addition of tetramethylpyrazine can increase the nutty and toasty aroma of the wine body (44), which contributes to the flavor of Chinese Baijiu and is also an important health functional factor. The abundance of acetolactate decarboxylase (EC 4.1.1.5) in summer-produced Daqu was significantly high, which contributes to the accumulation of acetoin and tetramethylpyrazine, and can improve the flavor of the wine. High temperature during fermentation favors the formation of tetramethylpyrazine (45), which was related to the significant enrichment of *Bacillus* in the summer-produced Daqu.

## 4 Conclusion

A total of 1,382 OTUs were obtained from 48 composite samples, 21 phyla, 529 genera, and 47 core bacterial genera were identified. Additionally, Pantoea, Bacillus, Lentibacillus, and Oceanobacillus were the representative functional bacterial genera of spring, summer, autumn, and winter, respectively. Functional bacteria were significantly enriched in the summer-produced Daqu, which contributed to improving the quality of JFDQ. Microbial community function prediction and differential metabolite pathway enrichment analysis revealed the functional differences of Daqu samples produced in different seasons, especially related to glycolysis, acetoin biosynthesis, and ethanol fermentation. Although the Daqu microbiota produced in the four seasons had a high degree of commonality, each had specific microbiota and functional metagenomic characteristics, suggesting that they have distinct but complementary roles in the fermentation process in different seasons. This study will guide the targeted control of microbial community succession and optimization of the quality in JFDQ.

## Data availability statement

The data presented in this study are deposited in the Sequence Read Archive (SRA) repository, accession number: PRJNA904081.

## Author contributions

YH: funding acquisition, resources, and methodology. LW: data curation and writing – original draft. YC: editing. XH: methodology and investigation. All authors contributed to the article and approved the submitted version.

## Footnotes

1
http://www.mothur.org/wiki

2
https://www.majorbio.com
