# Supplementary material for: Analysis of bacterial diversity and functional differences of Jiang-flavored Daqu produced in different seasons
Source: Front Nutr. 2023 Jan 4;9:1078132. doi: 10.3389/fnut.2022.1078132 (PMC9845603; doi:10.3389/fnut.2022.1078132)
Supplement: Supplementary file 1 [file Table_1.DOCX]

**Analysis of bacterial diversity and functional differences of Jiang-flavored Daqu produced in different seasons**

Lamei Wang^a,^ ^b, †^, Yuxin Cheng^a, †^, Xiaoxia Hu^c^, Yongguang Huang^a, b,^ *

^a^ College of Liquor and Food Engineering, Key Laboratory of Fermentation Engineering and Biological Pharmacy of Guizhou Province, Guizhou University, Guiyang, Guizhou, 550025, China

^b^ Key Laboratory of Fermentation Engineering and Biological Pharmacy of Guizhou Province

^c^ Guizhou Moutai Brewery (Group) Xijiu Co. Ltd., Xishui, Guizhou, 564622, China

^†^ These authors contributed equally to this work and were considered co-first authors.

* Correspondence: Prof. Yongguang Huang, Ph. D. College of liquor and food engineering, Guizhou University, Guiyang, People’s Republic of China;

Tel.: +86-0851-83651232; Fax: +86-0851-83651232;

E-mail: 772566120@qq.com; yghuang1@gzu.edu.cn

# Supplementary Tables

# Table S1. Microbial community richness and diversity indices of the 16S rRNA sequences for clustering at 97% sequence similarity from different seasons of Daqu.

| **Samples ID** | **Sobs** | **Shannon** | **Simpson** | **Ace** | **Chao 1** | **Coverage** |
| --- | --- | --- | --- | --- | --- | --- |
| Spring | 191.00 | 2.63 | 0.15 | 308.81 | 280.97 | 0.997 |
| Summer | 232.83 | 2.61 | 0.23 | 347.21 | 314.66 | 0.997 |
| Autumn | 216.75 | 2.90 | 0.11 | 332.93 | 308.13 | 0.997 |
| Winter | 239.50 | 2.93 | 0.11 | 350.90 | 326.52 | 0.997 |

## Table S2. The bacterial functions predicted by PICRUSt2, a total of 41 different level 3 KEGG metabolic pathways were predicted and screened (P < 0.05).

| **Level 1** | **Level 2** | **Level 3** |
| --- | --- | --- |
| Metabolism | Amino acid metabolism | Glycine, serine and threonine metabolism |
|  |  | Arginine biosynthesis |
|  |  | Lysine degradation |
|  |  | Valine, leucine and isoleucine biosynthesis |
|  | Biosynthesis of other secondary metabolites | Glucosinolate biosynthesis |
|  |  | Biosynthesis of various secondary metabolites - part 2 |
|  |  | Penicillin and cephalosporin biosynthesis |
|  |  | Caffeine metabolism |
|  |  | Stilbenoid, diarylheptanoid and gingerol biosynthesis |
|  |  | Flavonoid biosynthesis |
|  | Carbohydrate metabolism | Pyruvate metabolism |
|  |  | Pentose phosphate pathway |
|  |  | C5-Branched dibasic acid metabolism |
|  | Glycan biosynthesis and metabolism | Glycosaminoglycan degradation |
|  |  | Arabinogalactan biosynthesis - Mycobacterium |
|  | Lipid metabolism | Glycerophospholipid metabolism |
|  |  | alpha-Linolenic acid metabolism |
|  | Metabolism of cofactors and vitamins | Folate biosynthesis |
|  |  | Pantothenate and CoA biosynthesis |
|  |  | Thiamine metabolism |
|  | Metabolism of other amino acids | Phosphonate and phosphinate metabolism |
|  | Metabolism of terpenoids and polyketides | Polyketide sugar unit biosynthesis |
|  |  | Biosynthesis of type II polyketide products |
|  | Nucleotide metabolism | Pyrimidine metabolism |
|  | Xenobiotics biodegradation and metabolism | Aminobenzoate degradation |
|  |  | Ethylbenzene degradation |
|  |  | Steroid degradation |
|  | Energy metabolism | Photosynthesis - antenna proteins |
| Genetic  Information  Processing | Replication and repair | Fanconi anemia pathway |
|  | Folding, sorting and degradation | RNA degradation |
|  |  | Proteasome |
|  | Translation | RNA transport |
|  |  | mRNA surveillance pathway |
| Cellular  Processes | Cell growth and death | Necroptosis |
|  |  | Apoptosis |
|  |  | Meiosis - yeast |
|  |  | Apoptosis - multiple species |
|  |  | p53 signaling pathway |
|  | Cell motility | Bacterial chemotaxis |
|  | Cellular community - prokaryotes | Biofilm formation - Escherichia coli |
|  |  | Biofilm formation - Pseudomonas aeruginosa |

## Table S3. The description of enzymes in glycolysis, acetoin biosynthesis, and ethanol fermentation pathways according to the KEGG database.

| **EC number** | **Description** |
| --- | --- |
| **Glycolysis pathway** | |
| EC:5.4.2.2 | Phosphoglucomutase |
| EC:3.1.3.10 | Glucose-1-phosphatase |
| EC:2.7.1.1 | Hexokinase |
| EC:2.7.7.63 | Lipoate--protein ligase |
| EC:5.3.1.9 | Glucose-6-phosphate isomerase |
| EC:2.7.1.1 | 6-phosphofructokinase |
| EC:3.1.3.11 | Fructose-bisphosphatase |
| EC:4.1.2.13 | Fructose-bisphosphate aldolase |
| EC:1.2.1.12 | Glyceraldehyde-3-phosphate dehydrogenase (phosphorylating) |
| EC:2.7.2.3 | Phosphoglycerate kinase |
| EC:5.4.2.11 | Phosphoglycerate mutase (2,3-diphosphoglycerate-dependent) |
| EC:4.2.1.11 | Phosphopyruvate hydratase |
| EC:2.7.1.40 | Pyruvate kinase |
| **Acetoin biosynthesis pathway** | |
| EC:2.2.1.6 | Acetolactate synthase |
| EC:4.1.1.5 | Acetolactate decarboxylase |
| EC:1.1.1.304 | Diacetyl reductase ((S)-acetoin forming) |
| EC:1.1.1.303 | Diacetyl reductase ((R)-acetoin forming) |
| **Ethanol fermentation pathway** | |
| EC:1.2.4.1 | Pyruvate dehydrogenase (acetyl-transferring) |
| EC:2.3.1.12 | Dihydrolipoyllysine-residue acetyltransferase |
| EC:6.2.1.13 | Acetate--CoA ligase (ADP-forming) |
| EC:1.8.1.4 | Dihydrolipoyl dehydrogenase |
| EC:1.2.7.1 | Pyruvate synthase |
| EC:1.2.7.11 | 2-oxoacid oxidoreductase (ferredoxin) |
| EC:1.1.1.1 | Alcohol dehydrogenase |
| EC:1.1.1.2 | Alcohol dehydrogenase (NADP(+)) |

# Supplementary Figures

**
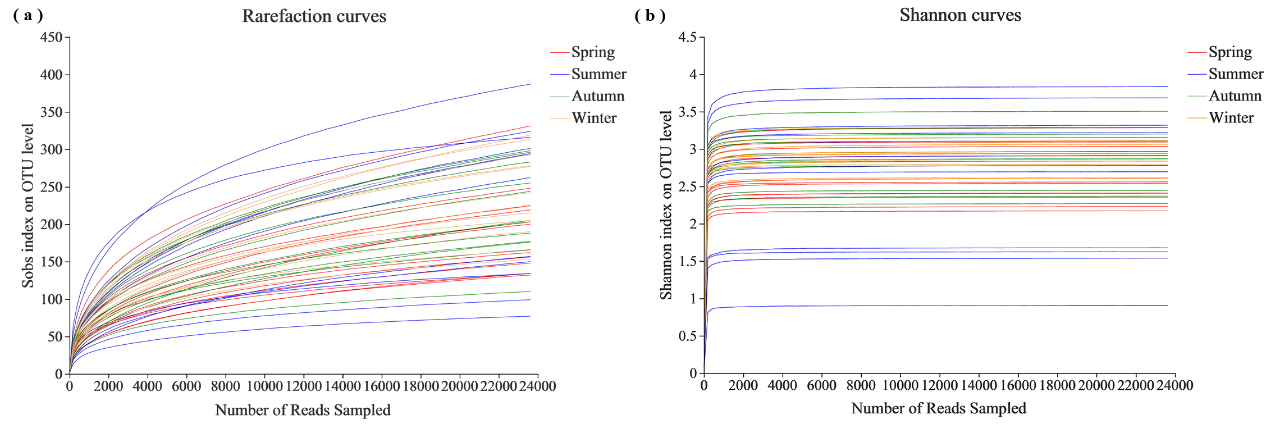
**

## Fig. S1. Rarefaction analysis of the samples. Rarefaction curves and Shannon curves of OTUs from different samples clustered at 97% sequence identity.
